# Overview on Current Trends and Emerging Therapies in the Chemotherapy of Patients with Human Epidermal Growth Factor Receptor 2-Positive Breast Cancer

**DOI:** 10.31661/gmj.v12i.3021

**Published:** 2023-08-20

**Authors:** Jian Qian, Yunxia Xu, Xiaokai Ling, Fenhua Wang

**Affiliations:** ^1^ Thyroid and Breast Surgery, Hangzhou Linping District Maternal and Child Health Care Hospital, Hangzhou 311199, Zhejiang, China

**Keywords:** HER2-positive, Breast Cancer, Chemotherapy, Targeted Therapies, Combination Treatment, Immune Checkpoint Inhibitors

## Abstract

Over the past few decades, significant progress has been made in the management of human epidermal growth factor receptor 2 (HER2)-positive breast cancer (BC) due to the development of targeted therapies. However, HER2-positive BC is an aggressive subtype, posing significant challenges, including treatment resistance and disease recurrence. Current standard treatment options for HER2-positive BC include combinations of chemotherapy drugs, targeted therapies such as trastuzumab and pertuzumab, and hormone therapies. However, some important limitations of these treatments, such as resistance and adverse effects, are reported. Also, we showed emerging therapeutic options, such as novel chemotherapy agents, antibody-drug conjugates, and immune checkpoint inhibitors, and discussed their mechanisms of action, potential benefits, and potential future directions in the field.

## Introduction

Breast cancer (BC) is one of the most prevalent malignancies worldwide, affecting millions of women annually [1][2]. Among the various subtypes of BC, human epidermal growth factor receptor 2 (HER2)-positive BC comprises a significant proportion, representing approximately 15-20% of cases [3]. The overexpression or amplification of the HER2 receptor leads to a more aggressive tumor phenotype, higher risk of recurrence, and poorer prognosis [4].

Significant progress has been made in the treatment landscape of HER2-positive BC over the past few decades [5]. However, de novo or acquired resistance to chemotherapy agents remains a challenge, necessitating the exploration of alternative treatment strategies [6].

Hence, in recent years, extensive research efforts have been dedicated to identifying new therapeutic approaches for patients with HER2-positive BC. This review aims to provide an overview of current trends and emerging therapies in the chemotherapy of HER2-positive BC and highlight the latest developments in targeted therapies, combination treatment strategies, immunotherapy, and potential future directions.

Targeted Therapies

Targeted therapies have revolutionized the treatment landscape for patients with HER2-positive BC [7]. The overexpression of the HER2 receptor plays a crucial role in the aggressive nature of this subtype, making it an attractive target for therapy [8]. The introduction of trastuzumab (known as Herceptin) [9], a monoclonal antibody targeting the extracellular domain of HER2, has significantly improved clinical outcomes and reduced the risk of recurrence and mortality.

In addition to trastuzumab, newer targeted therapies have emerged as promising treatment options for patients with HER2-positive BC. Trastuzumab emtansine (T-DM1), an antibody-drug conjugate-combines trastuzumab with a cytotoxic agent-allowing for targeted delivery of chemotherapy directly to HER2-positive tumor cells [10]. Clinical trials revealed that T-DM1 compared to traditional chemotherapy regimens, markedly improved progression-free survival (PFS) and overall survival (OS) rates [11][12].

Furthermore, small-molecule tyrosine kinase inhibitors (TKIs), such as neratinib and tucatinib, have demonstrated efficacy in overcoming resistance to trastuzumab and improving outcomes for patients with HER2-positive BC [13][14]. Neratinib irreversibly binds to the intracellular domain of HER2, inhibiting signaling pathways that promote tumor growth [15]. Clinical trials demonstrated that adding neratinib to trastuzumab-based therapy could improve disease-free survival rates in patients with early-stage HER2-positive BC [16][17].

Tucatinib is another small molecule TKI that selectively targets HER2 and has shown promising results in combination with trastuzumab and capecitabine chemotherapy [18][19]. Recent phase II and III clinical trials indicated improved PFS and OS among patients who received tucatinib plus standard therapy [20][21].

Moreover, the development of novel antibodies, including bispecific antibodies and antibody-drug conjugates, holds promise in further enhancing the efficacy of targeted therapies for HER2-positive BC [22]. Bispecific antibodies, such as trastuzumab deruxtecan (T-DXd; DS-8201), can simultaneously target HER2-positive tumor cells and immune cells, promoting immune-mediated cytotoxicity and enhancing anti-tumor responses [23]. These innovative treatment approaches have shown encouraging results in clinical trials and suggest potential new treatment options for patients with HER2-positive BC [24][25].

Immunotherapy

Immunotherapy has emerged as a promising treatment approach for patients with HER2-positive BC. It aims to attenuate immune system suppression and evasion of immune surveillance induced by cancer cells [26]. While immunotherapy has shown remarkable success in various malignancies [27][28], its application in HER2-positive BC is a relatively recent and evolving field [29].

One of the important targets of immunotherapy in HER2-positive BC is the programmed cell death-protein 1 (PD-1)/programmed death-ligand 1 (PD-L1) pathway, which plays an important role in immune system regulation and tumor immune evasion [30]. Immune checkpoint inhibitors, such as pembrolizumab, atezolizumab, and durvalumab, block the interaction between PD-1 on immune cells and PD-L1 on cancer cells, thereby permitting the immune system to attack the tumor cells [31][32][33].

Early clinical trials exploring the use of immune checkpoint inhibitors as monotherapy in patients with HER2-positive BC have shown modest response rates [34][35]. However, the combination of immune checkpoint inhibitors with HER2-targeted therapies or chemotherapy has shown more promising results [35]. Preclinical models and ongoing clinical studies suggest combining immunotherapy with HER2-targeted agents could enhance the anti-tumor immune response and improve clinical outcomes [36].

Another approach in immunotherapy for HER2-positive BC is the development of antibody-drug conjugates (ADCs), which combine the targeting ability of monoclonal antibodies with the cytotoxic effect of chemotherapy [37]. ADCs, such as T-DXd, deliver a potent chemotherapy agent directly to HER2-positive tumor cells, increasing tumor cell death [38]. Indeed, T-DXd has demonstrated encouraging activity in patients with HER2-positive BC that were previously treated with HER2-targeted therapies and chemotherapy, with promising response rates and durable responses [39].In addition to PD-1/PD-L1 inhibitors and ADCs, other immunotherapeutic strategies, such as cancer vaccines and adoptive cell therapy, are also being explored in HER2-positive BC [40][41]. Cancer vaccines stimulate the immune system by presenting specific antigens related to the HER2 protein, training the immune system to recognize and inhibit HER2-positive cancer cells [42]. Adoptive cell therapy involves genetically modifying a patient’s immune cells to express chimeric antigen receptors specifically targeting HER2-positive cancer cells [43]. These approaches are still in the early stages of development, but they are promising for improving treatment outcomes for patients with HER2-positive BC [44].

Combination Treatment Strategies

Combination treatment strategies have become increasingly important in the management of HER2-positive BC [45]. The integration of multiple treatment modalities aims to maximize therapeutic efficacy, overcome resistance, and improve patient outcomes [46]. Several approaches have been introduced to optimize combination therapies for patients with HER2-positive BC [47]. One of the critical strategies in combination therapy is the dual blockade of HER2 receptors [48]. This involves using multiple HER2-targeted agents to inhibit different aspects of the HER2 signaling pathway [49]. The combination of trastuzumab and pertuzumab, both monoclonal antibodies targeting different epitopes of HER2, indicated improved efficacy compared to trastuzumab alone [50]. Clinical trials have shown enhanced tumor responses and prolonged survival rates for patients with HER2-positive BC that received dual HER2 blockade [51][52].

Neoadjuvant and adjuvant studies demonstrated that adding chemotherapy to HER2-targeted agents further enhances treatment responses and reduces the risk of disease recurrence [53]. Furthermore, emerging evidence suggests that immune checkpoint inhibitors, such as PD-1 and PD-L1 inhibitors, may have a role in combination treatment strategies for HER2-positive BC [54].

Also, personalized medicine approaches, guided by genomic profiling and liquid biopsies, are being increasingly utilized to tailor combination treatment strategies for HER2-positive BC [55]. Biomarker analysis enables the identification of specific molecular alterations and the selection of targeted therapies most likely to be effective [56]. Integrating genomic profiling with combination treatment strategies could individualize treatment regimens and improve patient outcomes [57].

Future Directions

Future directions for HER2-positive BC involve advancements in personalized medicine, targeted therapies, and molecular profiling. One promising area of research is the development of more effective targeted therapies. Currently, HER2-positive BC is treated with drugs such as trastuzumab and pertuzumab, which target the HER2 protein specifically [58]. However, some patients develop resistance to these therapies over time [59]. Future research aims to identify additional genetic alterations and molecular targets that can be targeted along with HER2, to improve treatment outcomes and overcome resistance [60].

Also, advancements in molecular profiling techniques facilitated personalized medicine approaches in HER2-positive BC [61]. Researchers are developing methods to identify specific genetic mutations and alterations in patients’ tumors, allowing for more targeted and tailored treatment strategies [62]. This approach aims to optimize therapy, minimize side effects, and ultimately improve patient outcomes [63].

Furthermore, studies are ongoing to understand resistance mechanisms to current HER2-targeted therapies. By identifying the underlying causes of treatment resistance, clinicians could develop strategies to overcome it [64].

## Conclusion

The chemotherapy landscape for HER2-positive BC revealed several promising developments. The integration of targeted therapies, combination strategies, and immunotherapy has great potential to improve patient outcomes. Regarding the complexity of the disease, ongoing research and clinical trials are crucial to identify optimal treatment approaches and provide more effective, as well as, personalized therapies for patients with HER2-positive BC.

## Conflict of Interest

All the authors declare that there is any conflict of interests.
